# Post-Translational Regulation via Clp Protease Is Critical for Survival of *Mycobacterium tuberculosis*

**DOI:** 10.1371/journal.ppat.1003994

**Published:** 2014-03-06

**Authors:** Ravikiran M. Raju, Mark P. Jedrychowski, Jun-Rong Wei, Jessica T. Pinkham, Annie S. Park, Kathryn O'Brien, German Rehren, Dirk Schnappinger, Steven P. Gygi, Eric J. Rubin

**Affiliations:** 1 Department of Immunology and Infectious Diseases, Harvard School of Public Health, Boston, Massachusetts, United States of America; 2 Department of Cell Biology, Harvard Medical School, Boston, Massachusetts, United States of America; 3 Department of Microbiology and Immunology, Weill Cornell Medical College, New York, New York, United States of America; McGill University, Canada

## Abstract

Unlike most bacterial species, *Mycobacterium tuberculosis* depends on the Clp proteolysis system for survival even in *in vitro* conditions. We hypothesized that Clp is required for the physiologic turnover of mycobacterial proteins whose accumulation is deleterious to bacterial growth and survival. To identify cellular substrates, we employed quantitative proteomics and transcriptomics to identify the set of proteins that accumulated upon the loss of functional Clp protease. Among the set of potential Clp substrates uncovered, we were able to unambiguously identify WhiB1, an essential transcriptional repressor capable of auto-repression, as a substrate of the mycobacterial Clp protease. Dysregulation of WhiB1 turnover had a toxic effect that was not rescued by repression of *whiB1* transcription. Thus, under normal growth conditions, Clp protease is the predominant regulatory check on the levels of potentially toxic cellular proteins. Our findings add to the growing evidence of how post-translational regulation plays a critical role in the regulation of bacterial physiology.

## Author Summary

To date, studies on the regulation of physiology and virulence in *Mycobacterium tuberculosis* (Mtb) have focused on how transcriptional changes lead to adaptation. Interestingly, Mtb has numerous proteases that are essential for normal growth suggesting that protein turnover may also play an important regulatory role in the pathogen. We used novel methods to identify the set of proteins that are degraded by the essential Clp protease. The degradation of one protein, WhiB1, was required for normal growth confirming that inhibiting turnover of certain substrates can have a lethal effect. The understanding of essential pathways in Mtb will be important for the discovery of novel drugs to aid in the global fight against tuberculosis.

## Introduction

Our understanding of how bacteria regulate cellular processes has long focused on the role of transcription factors in the modulation of cellular responses. In eukaryotes, however, elucidation of the ubiquitin-proteasome pathway has illustrated that targeted degradation of functional proteins is often employed as a regulatory mechanism[1], [2]. Like eukaryotes, bacteria possess an array of compartmentalized proteolytic complexes, capable of degrading proteins into smaller polypeptides and amino acids[3], [4]. Initially, they were thought to maintain protein quality control through the recognition of misfolded, aberrant protein products. Several studies identified an array of endogenous proteins that were targeted for degradation in bacteria[5], [6]. While this suggested an active role of proteolysis in the regulation of bacterial physiology, it has been difficult to determine the functional significance of protein degradation by these proteolytic machines in bacteria.

*Mycobacterium tuberculosis* (Mtb), the causative agent of tuberculosis that kills nearly 1.3 million people annually[7], may provide unique insights into the importance of targeted protein degradation in bacteria. In most model prokaryotes, where the compartmentalized proteases have been extensively studied, they are largely dispensable for normal growth[8], [9]. However, a genome-wide screen for essential genes in Mtb suggested that numerous proteolytic complexes (namely Clp, FtsH, and HtrA) were absolutely required for cell survival, providing evidence for their critical role in bacterial physiology[10]. Further studies on the Clp complex revealed that inhibition or depletion of the protease results in mycobacterial death both *in vitro* and in a mouse model of infection[11], [12].

The ATP-dependent Clp proteolytic complex is composed of a serine proteolytic core that interacts with a set of regulatory ATPases. In mycobacteria, the ClpP proteolytic core, normally a homomeric complex in most bacteria, is actually comprised of two stacked heptameric rings of ClpP1 and ClpP2 multimers [13]. Targeted proteins enter through an axial pore that is regulated by the interaction of the ClpP1P2 complex with various AAA+ ATPases (ClpC1 and ClpX in Mtb) [14], [15], thus forming the full Clp complex. In Mtb, though endogenous protein substrates have yet to be identified, Clp has been implicated in the recycling of abnormal peptides stalled on the ribosome, through recognition of SsrA-tagged proteins[11].

In this study, we constructed a conditional ClpP1P2 protease mutant in Mtb and compared the proteomes of ClpP1P2-deficient cells to wildtype Mtb using recently developed MS3-based isobaric multiplexed quantitative proteomics. We identified one of the targets of the Clp protease as WhiB1, an essential transcriptional repressor that contains an iron-sulfur cluster. Blocking Clp-dependent degradation of WhiB1 resulted in stabilization of WhiB1 in mycobacteria. This stabilized allele was functional but toxic even at physiological levels, suggesting that proteolysis is the primary regulatory check on the amount of WhiB1 present. These data establish a mechanism for the essentiality of Clp protease in mycobacteria, and provide critical evidence of the dominant role that protein turnover plays in regulating bacterial physiology.

## Results

### Clp protease is essential in *Mycobacterium tuberculosis*

**Figure 1:**
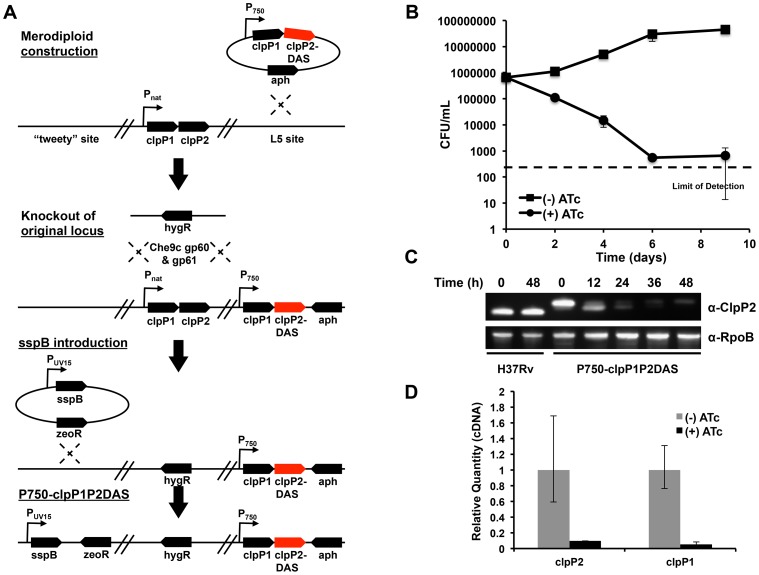
Clp protease is required for normal growth in *Mycobacterium tuberculosis*. (A) Schematic depicting the construction of a conditional clpP1P2 mutant (Mtb P750-clpP1P2DAS). A stable, chromosomal merodiploid was first constructed by integrating an additional copy of the clpP1P2 operon at the L5 site. In this additional operon, the native promoter was replaced with a ATc responsive promoter, and the end of clpP2 was modified by appending a DAS inducible degradation motif. Next, the original clpP1P2 operon was deleted using mycobacterial recombineering. Lastly, a plasmid bearing ATc-inducible sspB was integrated into the attB site on the chromosome, allowing for inducible degradation of ClpP2-DAS. (B) Growth curves of Mtb P750-clpP1P2DAS in the presence or absence of ATc (1.5 µg/mL). Data are represented as mean CFU/mL +/− standard deviation. (C) Depletion of ClpP2-DAS was tracked over time by immunoblot in wt H37Rv and P750-clpP1P2DAS in the presence or absence of ATc (1.5 µg/mL). Blots were probed with α-ClpP2 and α-RpoB (loading control). (D) Quantitative PCR to determine clpP1 and clpP2 transcript levels using RNA generated from Mtb P750-clpP1P2DAS after growth for 48 h in the presence or absence of ATc (1.5 µg/mL). Relative standard curves were generated for each probe set, and sigA transcript was used as an endogenous control. Data are represented as mean fold change, normalized to transcript in (−) ATc cultures +/− SEM of technical replicates.

The essentiality of Clp protease has been demonstrated in the non-pathogenic, fast growing model organism, *Mycobacterium smegmatis* (Msm), but not in *Mycobacterium tuberculosis* (Mtb). We constructed a Clp protease conditional mutant in Mtb that took advantage of complementary systems of promoter regulation and inducible protein degradation, recently developed for use in mycobacteria (**FIGURE 1A**). Addition of anhydrotetracycline (ATc) to this strain, denoted P750-clpP1P2DAS, simultaneously repressed transcription of the clpP1P2 operon and led to the degradation of existing ClpP2 protein[16].

At low inoculums (5×10^5^ CFU/mL), addition of ATc (1.5 µg/mL) to P750-clpP1P2DAS had a bactericidal effect, demonstrating that ClpP1 and ClpP2 are essential in Mtb (**FIGURE 1B**). At higher inocula (1×10^7^ CFU/mL), depletion also inhibited growth (**FIGURE S1**). These higher inocula allowed us to harvest cellular material for protein and transcript expression analysis. Production of SspB resulted in profound depletion of ClpP2-DAS within 48 hours, or two replicative cycles (**FIGURE 1C**). Furthermore, qPCR analysis of *clpP1* and *clpP2* mRNA revealed that by 48 hours, transcription at the *clpP1P2* locus was significantly repressed in cultures exposed to ATc (**FIGURE 1D**).

### Proteomic identification of Clp substrates in *Mycobacterium tuberculosis*

The P750-clpP1P2DAS strain enabled us to conditionally deplete the ClpP1P2 proteolytic core and explore the mechanism of essentiality of Clp protease in Mtb. We hypothesized that growth inhibition observed in this strain resulted from an accumulation of Clp substrates that were either toxic to the cell or that repressed normal growth. To identify potential substrates, we utilized LC/MS/MS3-based multiplexed quantitative proteomics with isobaric tandem mass tags (TMT) to quantify and compare the proteomes of Clp deficient and wildtype Mtb[17]. Briefly, six isobaric TMT (TMT126-131) tags (Thermo Fisher) have isobaric masses and are used to label the peptides from the proteomes of six different experimental conditions. When pooled, the same peptides from all conditions co-elute during fractionation. However, upon isolation and MS3 fragmentation of the initial single MS peptide peak, the six TMT molecules fragment differently between the 126–131 mass range. The resultant peak heights of the TMT ions represent the relative quantities of a given peptide between the different samples.

**Figure 2:**
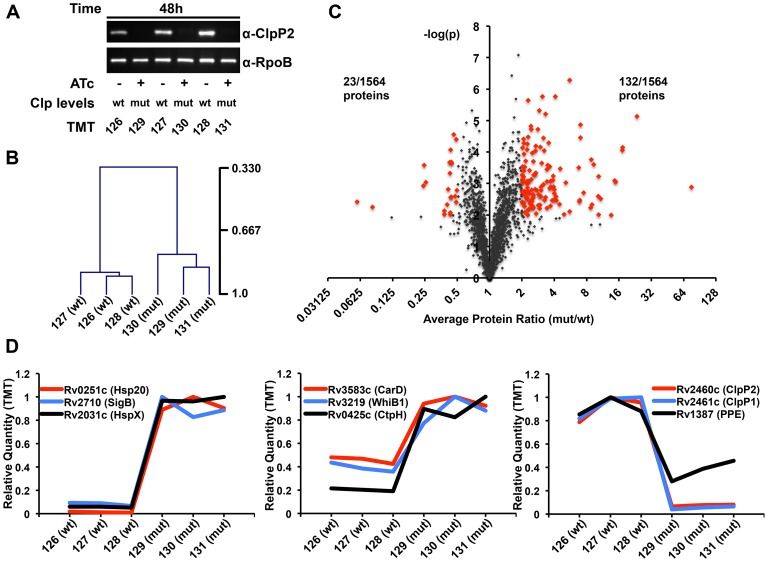
Proteomic profiling of P750-clpP1P2DAS in the presence and absence of ATc reveals a wide array of potential Clp protease substrates. (A) In triplicate, P750-clpP1P2DAS was grown for 48 hours in the absence, denoted “wt”, or presence of ATc (1.5 µg/mL), denoted “mut”, from a starting OD_600_ of 0.02. Immunoblotting of protein lysates with α-ClpP2 and α-RpoB (loading control) demonstrates degree of ClpP2-DAS depletion in mut cells. Samples were then used for TMT_6_ MS3-based quantitative proteomics. The specific TMT reagent used for each condition is listed under the immunoblot. (B) Normalized, summed intensities for all quantified proteins was used to perform Pearson correlational hierarchical clustering of biological replicates. (C) The Log2 ratio of average mutant protein intensity to average wildtype protein intensity plotted against the p-value determined by t-test, grouping the three biological replicates. The threshold for over-representation was set at an average ratio of greater than equal to 2, while the cut-off for under-representation was 0.5. In both instances, p-values below 0.01 were deemed significant. Proteins considered for further analysis are denoted in red. (D) The relative quantity of specific proteins plotted across the six TMT channels, for highly (left) and moderately (center) over-represented, and under-represented (right) proteins in the mutant versus wildtype conditions.

We harvested P750-clpP1P2DAS grown from an initial inoculum of 1×10^7^ CFU/mL for 48 hours either in the absence or presence of 1.5 µg/mL ATc, with biological triplicates (a total of six conditions and proteomic samples). Immunoblot analysis demonstrated a significant knockdown of ClpP2 in each of the cultures exposed to ATc (**FIGURE 2A**). Through TMT labeling followed by MS3-based quantitative proteomics[18], we quantified 1564 proteins. Hierarchical clustering using Pearson correlational analysis revealed a strong correlation between the proteomes of the three biological replicates (**FIGURE 2B**) [19]. A total of 132 proteins were significantly over-represented in mutant bacteria. We defined significant over-representation (or under-representation) as an average change of two-fold or more between mutant and wildtype conditions, and a p-value of less than or equal to 0.01 across the biological replicates (**FIGURE 2C**). Gene ontology (GO) analysis failed to reveal enrichment of any particular GO class among the over-represented proteins in Clp-depleted bacteria[20]. However, there were numerous transcriptional modulators with increased abundance in mutant bacteria (**TABLE S1**). Comparing protein intensity values between mutant and wildtype bacteria revealed a set of proteins that were highly over-represented (>5-fold increase, 24 proteins), moderately over-represented (2–5-fold increase, 108 proteins), or under-represented (>2-fold decrease, 23 proteins) (see examples in **FIGURE 2D**). ClpP1 and ClpP2 were the two most under-represented proteins in the screen, both depleted over 90% in mutant cells compared to wildtype.

### Transcriptional factors WhiB1 and CarD are likely Clp protease substrates

**Figure 3:**
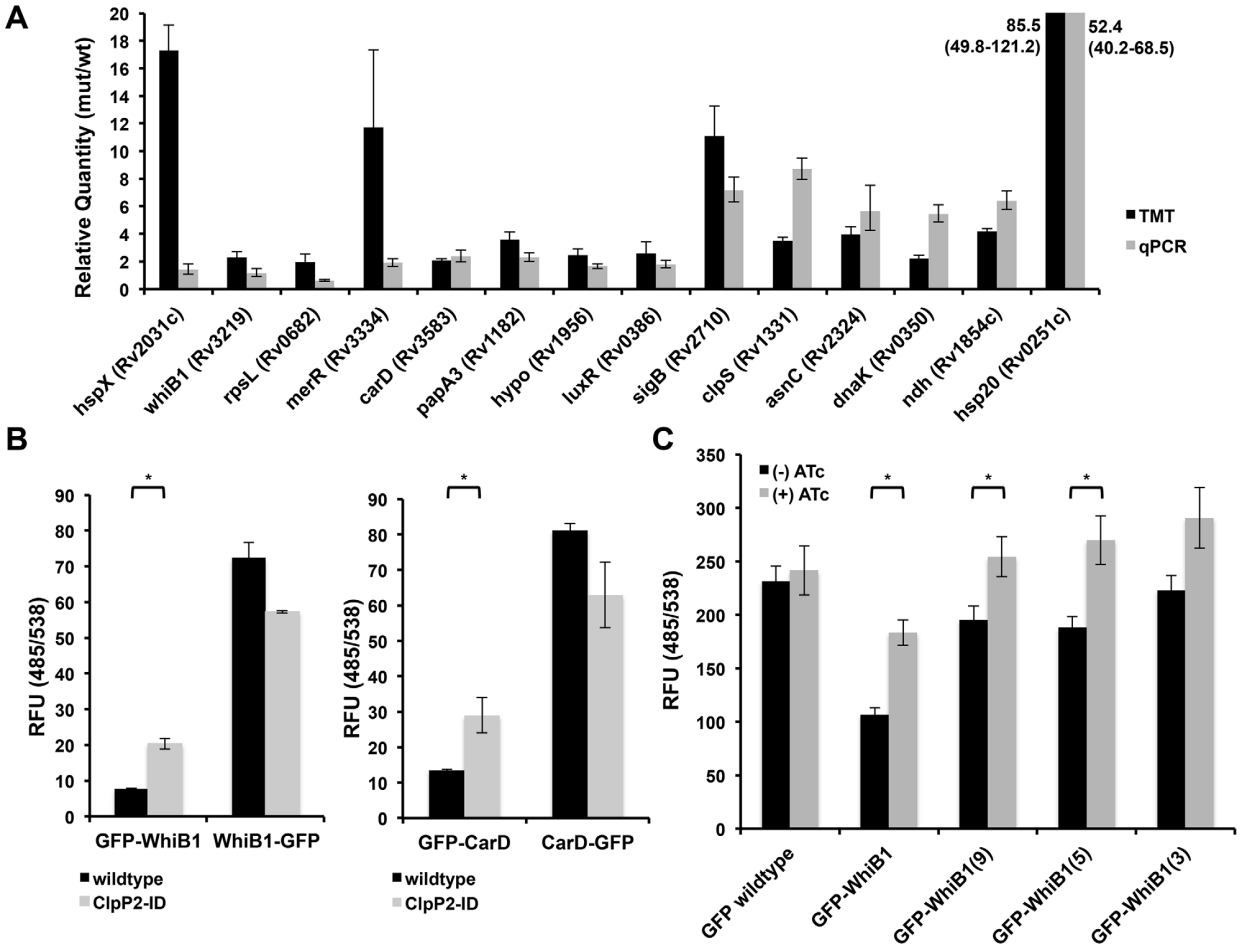
Validation of proteomic hits reveals that WhiB1 and CarD are likely Clp protease substrates. (A) Quantitative PCR to determine transcript levels of over-represented proteins upon depletion of Clp protease using RNA generated from Mtb P750-clpP1P2DAS after growth for 48 h in the presence or absence of ATc (1.5 µg/mL). Relative standard curves were generated for each probe set, and sigA transcript was used as an endogenous control. Data are represented as mean fold change, normalized to transcript in (−) ATc cultures +/− SEM of technical replicates. Protein ratios are derived from TMT experiments described in Figure 2, and represented as average ratios +/− standard deviation of biological replicates (B) Fluorescence (485/538) was measured for N- and C-terminal GFP fusions constructed for WhiB1 (left) and CarD (right), and induced for 8 hours in wildtype or clpP2-ID Msm with ATc (100 ng/mL). In clpP2-ID, ATc simultaneously induced fusion protein production and degradation of ClpP2. (C) Fluorescence (485/538) was measured for GFP fusions bearing a variable number of C-terminal residues from WhiB1 in clpP2-ID Msm, grown in the absence or presence of ATc (100 ng/mL) for 8 hours. In (B) and (C), data are represented as mean RFU +/− standard deviation of biological replicates. Asterisks denote a p-value <0.05 determined by t-test.

Protein accumulation upon Clp depletion could be the result of ineffective proteolysis due to reduced levels of Clp protease, but may also be due to a transcriptional upregulation of certain stress-induced proteins as a reaction to Clp depletion. The accumulation of numerous heat shock proteins suggested that, to some extent, this was the case. To determine the subset of over-represented proteins that were likely Clp substrates, we used quantitative PCR analysis to compare the transcript levels of putative substrates in Clp-deficient and wildtype bacteria. This analysis revealed three groups, one where increases in protein abundance upon Clp depletion could be explained by mRNA abundance, a second where there was a clear discordance between protein amount and transcript level change, and a third where the difference was less clear (**FIGURE 3A**). We posited that the latter two groups were more likely to contain Clp substrates, as the changes in protein abundance were more likely due to protein-level regulation than transcriptional upregulation.

To further validate potential substrates of the Clp protease, we turned to a conditional clpP2 mutant (clpP2_ID Msm) we had previously developed in Msm[11]. In this strain, an analogous protein degradation system leads to rapid loss of ClpP2 protein. We previously reported that this mutant allowed for rapid degradation of ClpP2, and ClpP2 depletion resulted in the accumulation of a reporter substrate due to decreased turnover. We performed similar quantitative proteomic and transcriptional analysis on this strain comparing proteins both with and without ClpP2 depletion (**FIGURE S2, TABLE S2**). Proteomic analysis revealed 107 proteins elevated in the Msm mutant compared to wildtype Msm, and a 9.3% overlap (n = 10 proteins) with the results from the Mtb screen (**TABLE S3**). Through our combined Mtb and Msm analysis, we identified two essential transcriptional effectors, CarD and WhiB1, with increased protein abundance and insignificant changes in transcript level between Clp-deficient and wildtype bacteria.

To assay WhiB1 and CarD degradation by Clp, we constructed GFP-fusion proteins by adding GFP to either the N- or C-terminus of each protein, and producing these fusions on an ATc-inducible promoter. These fusion proteins allowed us both to alter protease recognition by modifying a potential, terminal recognition sequence and follow the accumulation of the resultant protein. Additionally, by tightly regulating the transcription of these constructs on an inducible plasmid, we could prevent transcriptional modulation from confounding our results. Production in wildtype Msm revealed differential abundances, as measured by fluorescence, between N- and C-terminal fusions for each protein. (**FIGURE 3B** **, black bars**). Despite the differential fluorescence between the two WhiB1 fusions, quantitative PCR analysis revealed that inducible production of GFP-WhiB1 and WhiB1-GFP led to similar amounts of transcript in the cell, suggesting that differential fluorescence observed was regulated at the protein level (**FIGURE S3A**).

To demonstrate that this discrepancy was specifically due to Clp protease, we introduced the fusions into clpP2_ID Msm, where addition of ATc simultaneously induced production of each fusion construct and depletion of ClpP2, and assessed protein abundances. For both WhiB1 and CarD, depletion of ClpP2 resulted in an increase in the abundance of the N-terminal GFP fusion relative to wildtype Msm, as measured both by fluorometry and immunoblot (**FIGURE 3B** **, FIGURE S3B**), presumably reflecting stabilization due to reduced turnover. Several other proteins exhibited different effects. For both RpL28 and DnaA, C-terminal GFP fusions were actually less stable than their respective N-terminal constructs. In the case of RpL28, depletion of ClpP2 stabilized both fusions, suggesting that the motif for Clp recognition was internal and not dependent on an exposed terminus. DnaA fusions were not stabilized at all upon ClpP2 depletion suggesting that DnaA was either not a substrate or that both free ends were required for proteolysis (**FIGURE S4A**). From these results, it appears that there may be numerous recognition motifs that lead to Clp-dependent degradation. Unfortunately, bioinformatics analysis did not reveal any common motifs among the proteins identified as putative Clp substrates in our proteomic screening.

To test whether the C-terminus of WhiB1 was sufficient to confer destabilization and recognition by Clp protease we constructed a variety of fusions where a variable number of C-terminal WhiB1 residues were appended to the end of GFP. We found that the addition of the last fifteen, nine, and five amino acids of WhiB1 to GFP destabilized the protein with respect to wildtype GFP. Furthermore, wildtype levels of GFP were restored in these constructs upon ClpP2 depletion (**FIGURE 3C**). Similarly, the C-terminal fifteen residues of CarD destabilized GFP (**FIGURE S4B**).

### Stabilization of WhiB1 and blocking Clp-dependent degradation is toxic in mycobacteria

**Figure 4:**
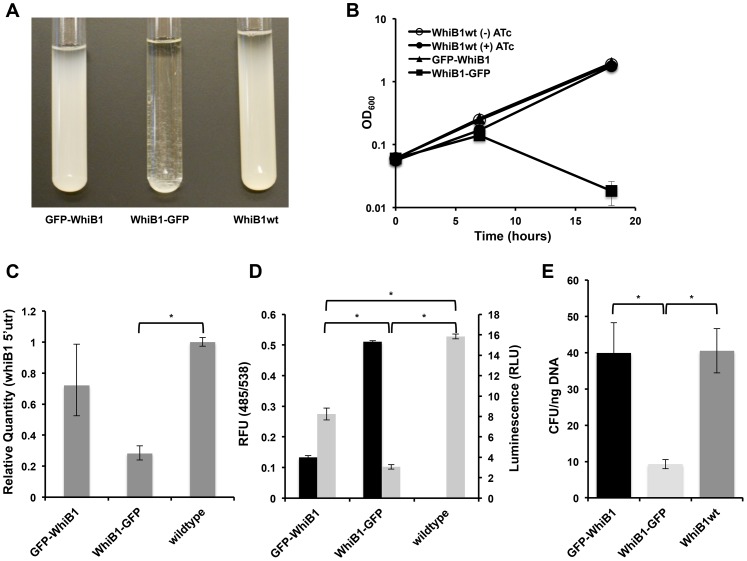
Blocking Clp-dependent degradation of WhiB1 is toxic in mycobacteria. (A) Inducible production of GFP-WhiB1, WhiB1-GFP, and WhiB1wt in Msm demonstrates growth inhibition of WhiB1-GFP producing bacteria. Over-production was induced with ATc (100 ng/mL). (B) Growth curves of strains producing WhiB1 constructs in the presence of ATc (100 ng/mL). As a control, strains producing WhiB1wt were grown in the absence of inducer. Data are represented as mean OD_600_ +/− standard deviation of biological replicates. (C) Quantitative PCR, using probe sets that hybridize to the whiB1 5′utr, to determine transcriptional repression at the endogenous *whiB1* locus in wildtype Msm, or Msm inducibly expressing *gfp-whiB1* or *whiB1-gfp*. RNA was isolated from cultures grown for 6 hours from a starting OD_600_ of 0.06 in the presence of the inducer ATc (100 ng/mL). Relative standard curves were generated for each probe set, and sigA transcript was used as an endogenous control. Data are represented as mean fold change, normalized to transcript in wildtype cultures +/− SEM of technical replicates. (D) Promoter reporters were constructed fusing the 500 bp upstream of whiB1 to luciferase. Luminescence (RLU, 100 ms exposure, grey bars) was measured in wildtype Msm, or Msm inducibly producing GFP-WhiB1 or WhiB1-GFP. Amounts of the fusion proteins were monitored by fluorescence (RFU, 485/538, black bars). Data are represented as RFU or RLU +/− standard deviation of biological replicates. (E) GFP whiB1 fusions and wildtype whiB1 were cloned into integrative plasmids, in which expression was driven by the native *whiB1* promoter. Constructs were transformed into Msm, and transformation efficiency was determined by calculating colony forming units (CFU) obtained per ng DNA. In (C), (D), and (E), asterisks denote a p-value <0.05, determined by t-test.

We noted that prolonged over-production of WhiB1-GFP inhibited the growth of mycobacteria and led to cell lysis (**FIGURE 4A and 4B**). This effect appeared to be specific to the C-terminal GFP fusion. To determine if the protein was still functional, and toxicity was not due to a non-specific effect, we determined if the WhiB1 fusion, as has been previously shown with wildtype WhiB1[21], could act as an auto-repressor. We used RT-PCR to determine transcription of the native protein in its normal chromosomal location, and found that the fusion protein was still able to serve as a repressor (**FIGURE 4C**). To further test the functionality of the WhiB1-GFP allele, we built a more sensitive reporter of promoter activity by fusing the putative whiB1 promoter to luciferase. By introducing this construct into strains inducibly producing the GFP fusions, we could simultaneously monitor fluorescence for protein abundance and stability and luminescence for *whiB1* promoter activity. For both fusions, the amount of repression appeared to correlate inversely with the amount of fusion protein present (**FIGURE 4D**). These observations suggest that the toxicity observed for WhiB1-GFP could be due to increased protein abundance, a result of stabilization from lack of recognition of the blocked C-terminus by Clp protease.

In all of the above experiments, the fusion *whiB1* constructs were expressed on multi-copy plasmids, perhaps leading to dramatic overexpression. To test if physiological levels of the degradation-deficient WhiB1 protein would be lethal, we constructed integrative plasmids with the native whiB1 promoter upstream of each fusion gene. This construct would lead to physiological levels of WhiB1 under natively regulated conditions. Transformation of these plasmids into Msm resulted in significantly lower transformation efficiencies for the plasmid bearing WhiB1-GFP compared to those with GFP-WhiB1 and WhiB1wt (**FIGURE 4E**). Colonies that resulted from WhiB1-GFP transformations plasmid were significantly smaller and took nearly twice as long to be seen than the GFP-WhiB1 or control WhiB1wt transformants. This suggests a considerable fitness cost associated with even a single copy of the stabilized allele is regulated by the native promoter.

## Discussion

Clp proteases have two primary functions. Like other degradative proteases, they play a role in protein quality control, degrading improperly synthesized or folded proteins. Indeed, this does appear to be the case in mycobacteria[11]. However, Clp proteases in other species also play an important regulatory role in degrading endogenous proteins. In other bacteria, identifying such Clp substrates has been facilitated by the use of *in vitro* systems in which Clp components have been inactivated so that binding could be assayed in the absence of proteolysis[22]. Thus far, due to the heteromeric composition of Clp in Mtb and its stringent requirements for normal growth, we have been unable to use an analogous approach. Instead, we relied on an *in vivo* assay for the accumulation of substrates taking advantage of recently developed, highly accurate proteomic methods. This approach is somewhat problematic as depleting Clp is quite toxic and it can be difficult to disentangle protein accumulation due to cellular responses from that resulting from lack of proteolysis of direct substrates. Nevertheless, using a combination of transcriptional analysis and sequence modifications to alter protein recognition, we were able to unambiguously define some new Clp protease substrates.

Alternative approaches to identifying ClpP substrates have been highly successful. For example, using an inactive ClpP mutant in *Staphylococcus aureus*, Feng, et al., [23] were able to affinity purify substrates. Unfortunately, despite many attempts, we were unable to use this method in mycobacteria. And, since protein abundance can be regulated both by transcription and degradation, using transcriptional regulation to screen for likely substrates will exclude some actual substrate proteins. For example, while CarD is not transcriptionally upregulated in Msm, it is mildly upregulated in Mtb; yet, we find that it is a substrate.

Thus far, using any approach has not clearly identified strong consensus sequences for degradation. In *E. coli*, the N end rule governs degradation of some substrates[24] others have conserved C terminal di-alanines[22] but most have none. Certainly, among the proteins we have found to be potential substrates we cannot identify a consensus.

Comparing degradation in two different mycobacterial species helps provide some additional confidence in recognized substrates. Although we used different regulatory approaches to deplete proteolytic subunits, we expect to have a good deal of biological concordance. ClpP1 and ClpP2 are both necessary for proteolysis to occur[11]. Depleting either or both should have similar effects. Our results suggest that there are significant similarities among substrates and, for example, both WhiB1 and CarD can be degraded by Clp in both species.

Do these results help us to understand why Clp is essential for bacterial growth and survival? Certainly, altering the sequence of WhiB1 so that it is no longer easily degraded results in cellular toxicity. Even when this stabilized allele is expressed at physiologic levels, it appears to be quite toxic. Thus, part of the function of Clp is to degrade the WhiB1 protein and keep its levels in check. This is reminiscent of the situation in *Caulobacter crescentus*, one of the few bacteria where Clp protease is also essential. In this organism, degradation of CtrA by Clp is absolutely necessary for the transition of a non-replicating, motile swarmer cell into a replicating, stalked cell[25], and loss of Clp protease activity results in cellular growth arrest. In mycobacteria, we can further establish the importance of post-translational regulation in prokaryotes by showing that WhiB1 levels are coordinated by a mixture of transcriptional and protein-level regulation, but that it is the interruption of protein-level regulation through Clp inhibition that is essential.

Why could the turnover of WhiB1 be required for normal growth? In mycobacteria, WhiB proteins are capable of binding redox-sensitive [4Fe-4S] clusters, which can serve as redox-active co-factors or as switches that reflect the reductive and oxidative potential of a cell[26]. The WhiB proteins may be disulfide reductases[27], but are certainly transcription factors capable of modulating cellular processes that are intimately tied to the redox state of the cell or perceived oxidative stress[28]. In Mtb, WhiB1 is an essential DNA binding protein capable of auto-repressing its own transcription[21]. ChIP-Seq to determine the WhiB1 regulon in Mtb has been undertaken, and preliminary data suggests the presence of 71 binding sites for WhiB1, thirteen of which are essential[29]. Accumulation of WhiB1 might repress at several sites resulting in the lack of synthesis of critical metabolites such as heme and riboflavin. Alternatively, supraphysiologic levels of WhiB1, due to absence of turnover, may result in binding of the transcription factor to low affinity sites with repression of other essential genes.

Does stabilization of WhiB1 account for all of the essentiality of Clp? Several lines of evidence suggest that this is unlikely to be true. Clp is the primary proteolysis system for degrading SsrA-tagged proteins, which result from trans-translation due to ribosomal stalling[30]. We have found that the small RNA, tmRNA, required for producing this tag, is itself essential[31]. In fact, this trans-translation system is at least one of the targets of the antimycobacterial drug pyrazinamide[32]. Loss of Clp would result in accumulation of these tagged proteins. Moreover, not only are the ClpP1 and ClpP2 protease subunits required for optimal *in vitro* growth but two of the alternate adapter proteins, ClpX and ClpC1 are essential as well. The fact that both these adapters are required supports the hypothesis that there are multiple substrates that need to be recognized (at least one per ATPase) by Clp protease to facilitate normal growth. For example, it is interesting to note that CarD has been implicated in the stringent response, and directly interacts with the beta-subunit of the RNA polymerase to down regulate transcription of the translational machinery and amino acid biosynthetic enzymes[33]. While stabilization of CarD alone is not sufficient to cause toxicity in mycobacteria, the transcriptional repression of enzymes important for vegetative growth facilitated by CarD may contribute to the growth inhibition observed upon depletion of Clp protease, and partially explain the essentiality of Clp protease in mycobacteria.

Transcriptional regulation plays a critical role in bacterial adaptation to new environments. However, much of regulation is likely to be post-transcriptional and no less important for bacterial survival. In the case of WhiB1, *M. tuberculosis* employs both transcriptional and post-transcriptional (Clp-mediated proteolysis) forms of regulation. Clearly, protein degradation mediated by Clp is required even in the absence of clear environmental stressors. Identifying the substrates for the Clp protease and other essential degradative proteases will help move us towards a more holistic understanding of how bacteria coordinate critical cellular activities through the integration of transcriptional and proteolytic regulation.

## Materials and Methods

### Bacterial strains and plasmids

Msm mc^2^155 (Msm) or Mtb H37Rv were grown at 37°C in Middlebrook 7H9 broth with 0.05% Tween 80 and ADC (0.5% BSA, 0.2% dextrose, 0.085% NaCl, 0.003 g catalase/1 L media). Mtb was additionally supplemented with oleic acid (0.006%). For growth curves, overnight cultures were diluted into the appropriate media and growth was either measured by OD_600_ or colony forming units per mL. A complete list of plasmids and primers used in this study can be found in **TABLE S4** and **TABLE S5**. Detailed procedures on strain construction can be found in the Supplemental Experimental Procedures.

### Fluorescence and luminescence measurements

In order to measure the abundance of GFP fusion proteins, cultures within one experiment were normalized based on OD_600_ values, spun down to remove media, and resuspended in 100 µL of PBS in a clear bottom 96 well plate. For luminescence, cultures were normalized based on OD_600_ values, and 150 µL of culture was used for measuring luciferase activity. 50 µL of Cell Culture 5X Lysis Reagent (Promega) was added to cultures, and samples were agitated for 10 min on an orbital shaker, at room temperature. Next, 75 µL of Luciferase Assay Substrate (Promega) was added to each sample and directly taken for measurement. Fluorescence was measured at 485/538 nm, and luminescence was measured at an exposure time of 10 milliseconds, by the Fluroskan Ascent FL plate reader (ThermoScientific). Results represent the median +/− standard deviation of biological replicates.

### Quantitative PCR

In Mtb and Msm, RNA was generated from equivalent of 20 mLs of cells at OD_600_ 0.5. Cultures were spun down, and subjected to bead beating (3X 45 sec each, 5 min on ice between pulses) after resuspension in TRIzol. After chloroform phase separation, genetic material was precipitated with isopropanol, resuspended in dH_2_O, and RNA was purified using RNeasy Mini Kit (Qiagen). To ensure no contamination from genomic DNA, purified RNA was subjected to an additional round of DNase digestion using the TURBO DNA-free Kit (Invitrogen). cDNA was created from isolated equal concentrations of RNA with the SuperScript III First Strand Synthesis System (Invitrogen). Quantitative PCR was performed with the SYBR FAST qPCR kit (KapaBiosystems) using the Applied Biosystems 7500 Fast Real-Time PCR System. All experiments were done using biological replicates, and representative experiments are depicted +/− standard error of mean of technical replicates.

### Sample preparation, protein digestion, and peptide TMT labeling

For Mtb proteomics, P750-clpP1P2DAS was diluted to a starting OD_600_ 0.02 in 900 mLs of 7H9 media. This culture was split into six 150 mL cultures, and 1.5 µg/mL ATc was added to three batches, while three were left to grow without induction. After 48 hours, cultures were spun down (10 min, 4000 rpm, 4°C) and washed 3X with PBS. Cultures were then resuspended in 1 mL Urea Lysis Buffer (8 M urea in 50 mM Tris pH 8.2, 75 mM NaCl, 50 mM NaF, 50 mM β-glycerophosphate, 1 mM Na orthovanadate, Roche Complete EDTA-free Protease Inhibitor Cocktail tablets), and subjected to bead beating (3X 45 sec each, 5 min on ice between pulses). Cell lysates were spun down (10 min, 13,000 rpm, 4°C), and samples were reduced with DTT (final concentration of 5 mM) for 30 min at 37°C and cooled to room temp for 15 minutes. Samples were then alkylated with iodoacetamide (final concentration of 14 mM) for 30 min at room temperature in the dark. Adding an additional 5 mM DTT and incubating samples at room temperature in the dark for 15 min quenched excess iodoacetamide. To remove samples from the BL3-level facility, proteins were precipitated with 20% trichloroacetic acid and incubated on ice for a hour. Proteins were pelleted by centrifugation (30 min, 13,000 rpm, 4°C), and pellets were washed twice with acetone. Samples were resuspended in 8 M urea containing 50 mM TRIS pH 8.5, diluted, and protein amounts were quantified using a BCA assay (ThermoScientific).

As the proteomics screen in Msm was performed prior to the development of MS3-based proteomics for TMT analysis, we performed MS2-based quantitation of TMT peptide signals. For Msm proteomics, Msm/pTet(OR)::HIV2pr and clpP2_ID Msm were diluted to a starting OD_600_ 0.05 in 450 mLs. ATc (100 ng/mL) was added to each strain, and cultures were divided into 150 mL batches. Samples were harvested at 0 h, 5 h, and 11 h post addition of ATc, spun down (10 min, 4000 rpm, 4°C), and washed 3X with PBS. Protein samples were prepared in a similar fashion as above, except no TCA precipitation was needed, and protein quantitation by the BCA assay was done prior to sample reduction and alkylation.

Proteins isolated from Mtb and Msm were digested overnight with Lys-C (Wako) in a 1∶100 enzyme:protein ratio in 4 M urea and 50 mM Tris-HCl (pH 8.2). Digests were acidified with formic acid to a pH of ∼2–3, and subjected to C_18_ solid-phase extraction (Sep-Pak, Waters). Isobaric labeling of the peptides was accomplished with sixplex TMT reagents (Thermo Scientific). Reagents (0.8 mg) were dissolved in 40 µl acetonitrile, and 20 µl of the solution was added to 200 µg of peptides dissolved in 100 µl of 50 mM HEPES (pH 8.5). After 1 h at room temperature, the reaction was quenched by adding 8 µl of 5% hydroxylamine for 15 minutes. Half of each of the labeled reactions was pooled into one vial, acidified with formic acid, diluted ACN to 5% final volume and subjected to C_18_ solid-phase extraction. Details on sample fractionation, liquid chromatography electrospray ionization tandem mass spectrometry, and data processing/analysis can be found in the Supplemental Experimental Procedures.

## Supporting Information

Figure S1 **Depletion of Clp protease in Mtb P750-clpP1P2DAS.** Growth curves of Mtb P750-clpP1P2DAS in the presence or absence of ATc (1.5 µg/mL) starting at a high initial inoculum (1×10^7^ CFU/mL). Higher inoculums facilitated protein and RNA extraction for proteomic and qPCR analysis, respectively. Data are represented as mean OD_600_ +/− standard deviation.
(PDF)

Figure S2 **Proteomic profiling of clpP2-ID Msm in the presence and absence of ATc reveals a set of potential Clp protease substrates.** (A) clpP2-ID Msm was grown in the presence or absence of ATc (100 ng/mL) from a starting OD_600_ of 0.04 for 5 or 11 hours. Depletion of ClpP2-ID was tracked by immunoblot of protein lysates, probing for α-myc and α-RpoB (loading control). Samples were then used for TMT_6_ MS2-based quantitative proteomics. The specific TMT reagent used for each condition is listed under the immunoblot. (B) Normalized, median intensities for all quantified proteins was used to perform Perason correlational hierarchical clustering of the different conditions. (C) The Log2 ratios of median protein intensity at 5 h for ClpP2 depleted cells (mut) to ClpP2 containing cells (wt). The threshold for over-representation was set at an average ratio of greater than or equal to 2, while the cut-off for under-representation was less than or equal to 0.5. Hits are denoted in red. (D) For a given set of proteins, the ratio of mutant to wildtype protein at 5 hours was compared to the ratio of transcript levels. Quantitative PCR was employed to determine transcript levels using RNA generated from clpP2-ID Msm after growth for 5 h in the presence or absence of ATc (100 ng/mL). Relative standard curves were generated for each probe set, and sigA transcript was used as an endogenous control. For each target, data are represented as mean fold change, of mutant cells normalized to wildtype transcript amount +/− SEM of technical replicates.
(PDF)

Figure S3 **Overproduction of WhiB1 fusion constructs (GFP-WhiB1 and WhiB1-GFP) confirmed by quantitative PCR and immunoblot.** (A) Quantitative PCR using a probe set that hybridized to both chromosomal and episomal copy of whiB1 to determine transcript abundance of whiB1 in strains inducibly over-expressing *gfp-whiB1* and *whiB1-gfp*, compared to wildtype Msm. RNA was isolated from cultures grown for 6 hours from a starting OD_600_ of 0.06 in the presence of the inducer ATc (100 ng/mL). Relative standard curves were generated for each probe set, and sigA transcript was used as an endogenous control. Data are represented as mean fold change, normalized to transcript in wildtype cultures +/− SEM of technical replicates. (B) Wildtype (wt) and clpP2-ID Msm inducibly producing WhiB1 GFP fusion proteins were grown in the presence ATc (100 ng/mL) from a starting OD600 of 0.04 for 9 hours to induce production of the fusion proteins. In the case of clpP12-ID, ATc simultaneously resulted in depletion of ClpP2. Accumulation of fusion proteins and depletion of ClpP2 were monitored by immunoblot using α-GFP and α-ClpP2, respectively.
(PDF)

Figure S4 **Characterization of DnaA, RpL28 as potential substrates of Clp protease, and localization of CarD degron.** (A) N- and C-terminal GFP-fusions for DnaA and RpL28, identified as potential Clp substrates from proteomic profiling of clpP2-ID Msm. Fluorescence (485/538) was measured for N- and C-terminal GFP fusions constructed for DnaA (left) and RpL28 (right), and induced for 8 hours in wildtype or clpP2-ID Msm with ATc (100 ng/mL). In clpP2-ID, ATc simultaneously induced fusion protein production and degradation of ClpP2. (B) Fluorescence (485/538) was measured for wildtype GFP, and GFP fusions bearing either the N-terminal C-terminal 15 amino acids from CarD. The constructs were expressed on a constitutive, episomal plasmid in wildtype Msm. In both (A) and (B), data are represented as mean RFU +/− standard deviation of biological replicates. Asterisks denote a p-value <0.05 determined by t-test.
(PDF)

Table S1 **Complete list of proteins identified through quantitative proteomic profiling of P750-clpP1P2DAS Mtb in the presence and absence of ATc.** List of all proteins identified through proteomic profiling of P750-clpP1P2DAS Mtb in the presence and absence of ATc are listed with the following data included: NCBI protein accession number, Rv number, Protein definition, Number of peptides identified, Normalized summed TMT intensity for wildtype replicates (Reporter ions 126, 127, 128), Normalized summed TMT intensity for Clp depleted replicates (Reporter ions 129, 130, 131), Average wildtype reporter ion intensity, Average mutant reporter ion intensity, mutant:wildtype ratio, and p-value (as determined by unpaired t-test grouping wildtype and mutant replicates).
(XLSX)

Table S2 **Complete list of proteins identified through quantitative proteomic profiling of clpP2-ID Msm in the presence and absence of ATc.** List of all proteins identified through quantitative proteomic profiling of clpP2-ID Msm in the presence and absence of ATc with the following data included: NCBI protein accession number, Protein definition, Number peptides identified, Normalized median TMT intensity for wildtype Msm at 0 h (Reporter ion 126), 5 h (Reporter ion 127), 11 h (Reporter ion 128), Normalized median TMT intensity for Clp depleted strain at 0 h (Reporter ion 129), 5 h (Reporter ion 130), 11 h (Reporter ion 131), Mutant:wildtype ratio at 0 h, Mutant:wildtype ratio at 5 h, and Mutant:wildtype ratio at 11 h.
(XLSX)

Table S3 **List of orthologous proteins in Mtb and Msm found through proteomic screening for Clp protease substrates.** Potential Clp substrates identified through Mtb and Msm screens were cross-referenced and 10 orthologous proteins were identified as potential Clp substrates in both species. Potential substrates in Mtb were defined as proteins that showed a greater than two-fold over-abundance in Clp-depleted strains compared to wildtype strains, with a p-value of less than 0.01 as measured by t-test grouping replicates. Potential substrates in Msm were defined as proteins that showed no difference prior to Clp depletion, but then showed a greater than two-fold overabundance in the Clp-depleted strain compared to the wildtype strain after 5 hours of depletion.
(XLSX)

Table S4 **Plasmids used in this study.** All plasmids used for experiments are listed, with specific references to particular plasmids included in the Supplementary Methods S1.
(DOC)

Table S5 **Primers used in this study.** All primers used for strain construction and qPCR are listed, with specific references to particular primers included in the Supplementary Methods S1.
(DOC)

Methods S1 **Supplementary Methods S1 includes further information on (1) Bacterial strains and plasmids, (2) Sample fractionation by high pH reverse phase and strong cation exchange chromatography, (3) Liquid chromatography electrospray ionization tandem mass spectrometry, and (4) Data processing: MS2 spectra assignment, data filtering and quantitative data analysis.**
(DOC)
