# Supplementary figures and images for: Post-Translational Regulation via Clp Protease Is Critical for Survival of Mycobacterium tuberculosis
Source: PLoS Pathog. 2014 Mar 6;10(3):e1003994. doi: 10.1371/journal.ppat.1003994 (PMC3946367; doi:10.1371/journal.ppat.1003994)

A

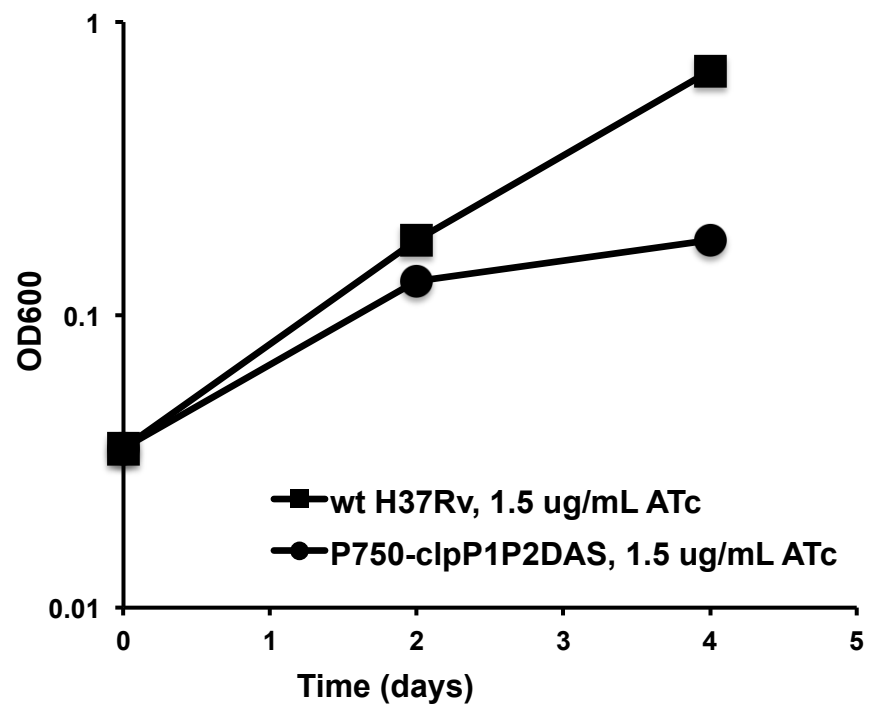

Supplement: Figure S1 — Depletion of Clp protease in Mtb P750-clpP1P2DAS. Growth curves of Mtb P750-clpP1P2DAS in the presence or absence of ATc (1.5 µg/mL) starting at a high initial inoculum (1×10^7^ CFU/mL). Higher inoculums facilitated protein and RNA extraction for proteomic and qPCR analysis, respectively. Data are represented as mean OD600 +/− standard deviation. (PDF) [file ppat.1003994.s001.pdf]

**A**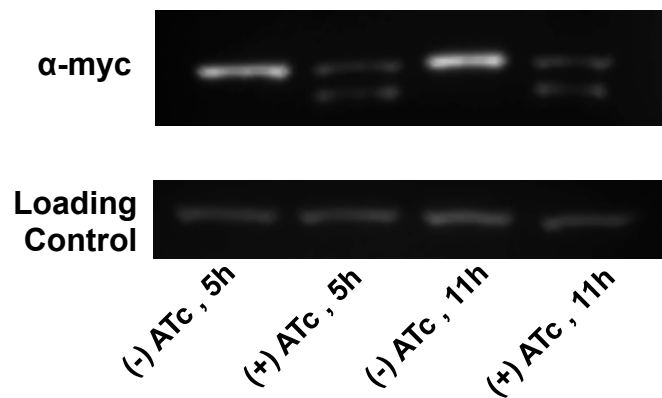**B**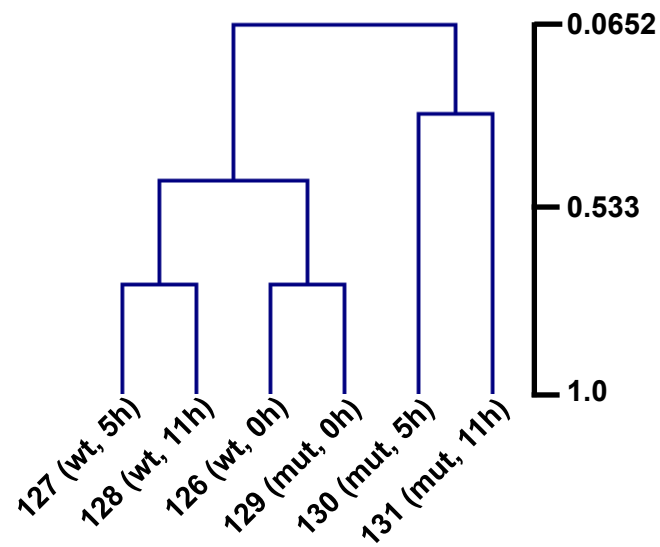**C**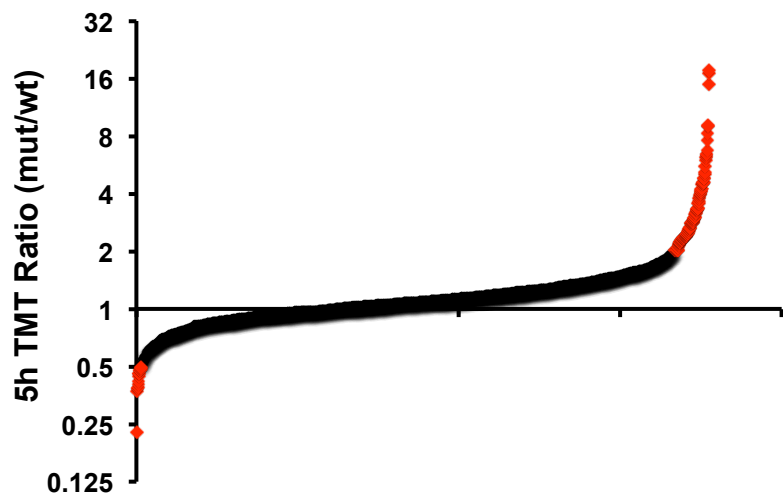**D**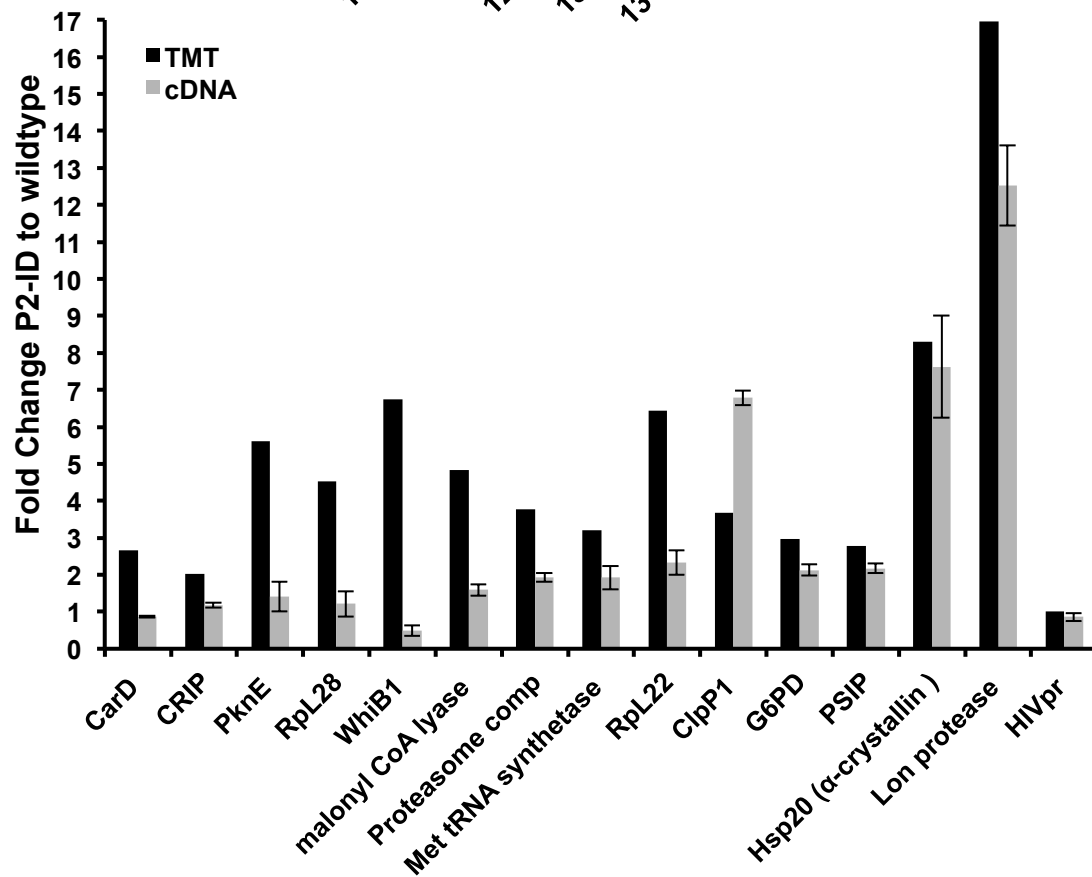

Supplement: Figure S2 — Proteomic profiling of clpP2-ID Msm in the presence and absence of ATc reveals a set of potential Clp protease substrates. (A) clpP2-ID Msm was grown in the presence or absence of ATc (100 ng/mL) from a starting OD600 of 0.04 for 5 or 11 hours. Depletion of ClpP2-ID was tracked by immunoblot of protein lysates, probing for α-myc and α-RpoB (loading control). Samples were then used for TMT6 MS2-based quantitative proteomics. The specific TMT reagent used for each condition is listed under the immunoblot. (B) Normalized, median intensities for all quantified proteins was used to perform Perason correlational hierarchical clustering of the different conditions. (C) The Log2 ratios of median protein intensity at 5 h for ClpP2 depleted cells (mut) to ClpP2 containing cells (wt). The threshold for over-representation was set at an average ratio of greater than or equal to 2, while the cut-off for under-representation was less than or equal to 0.5. Hits are denoted in red. (D) For a given set of proteins, the ratio of mutant to wildtype protein at 5 hours was compared to the ratio of transcript levels. Quantitative PCR was employed to determine transcript levels using RNA generated from clpP2-ID Msm after growth for 5 h in the presence or absence of ATc (100 ng/mL). Relative standard curves were generated for each probe set, and sigA transcript was used as an endogenous control. For each target, data are represented as mean fold change, of mutant cells normalized to wildtype transcript amount +/− SEM of technical replicates. (PDF) [file ppat.1003994.s002.pdf]

**A**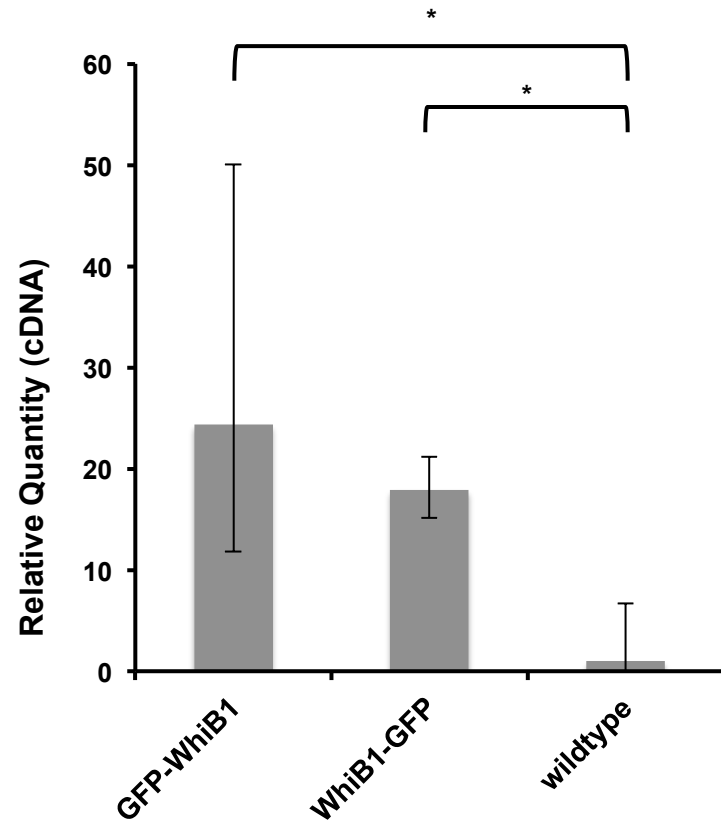**B**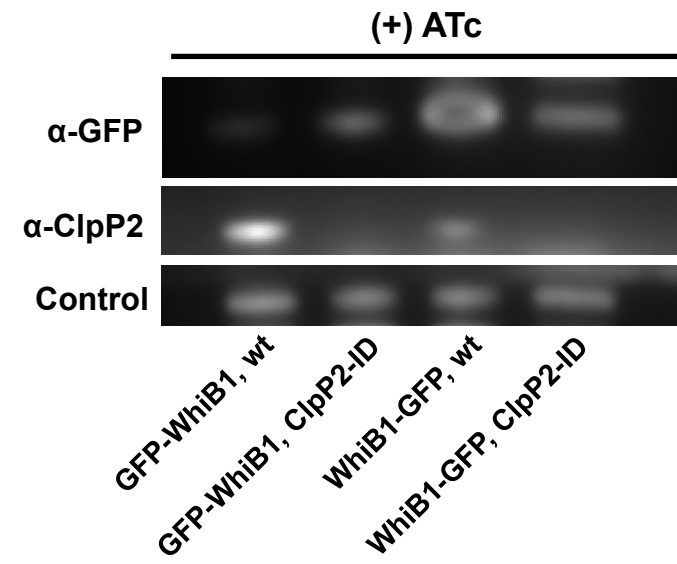

Supplement: Figure S3 — Overproduction of WhiB1 fusion constructs (GFP-WhiB1 and WhiB1-GFP) confirmed by quantitative PCR and immunoblot. (A) Quantitative PCR using a probe set that hybridized to both chromosomal and episomal copy of whiB1 to determine transcript abundance of whiB1 in strains inducibly over-expressing gfp-whiB1 and whiB1-gfp, compared to wildtype Msm. RNA was isolated from cultures grown for 6 hours from a starting OD600 of 0.06 in the presence of the inducer ATc (100 ng/mL). Relative standard curves were generated for each probe set, and sigA transcript was used as an endogenous control. Data are represented as mean fold change, normalized to transcript in wildtype cultures +/− SEM of technical replicates. (B) Wildtype (wt) and clpP2-ID Msm inducibly producing WhiB1 GFP fusion proteins were grown in the presence ATc (100 ng/mL) from a starting OD600 of 0.04 for 9 hours to induce production of the fusion proteins. In the case of clpP12-ID, ATc simultaneously resulted in depletion of ClpP2. Accumulation of fusion proteins and depletion of ClpP2 were monitored by immunoblot using α-GFP and α-ClpP2, respectively. (PDF) [file ppat.1003994.s003.pdf]

**A**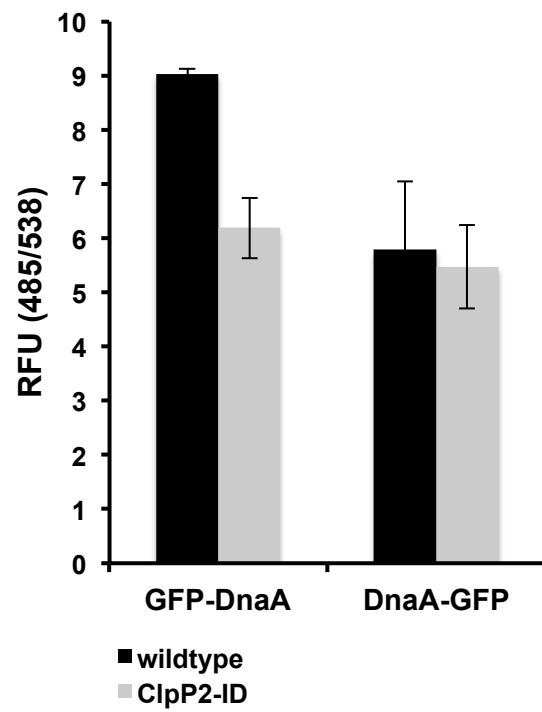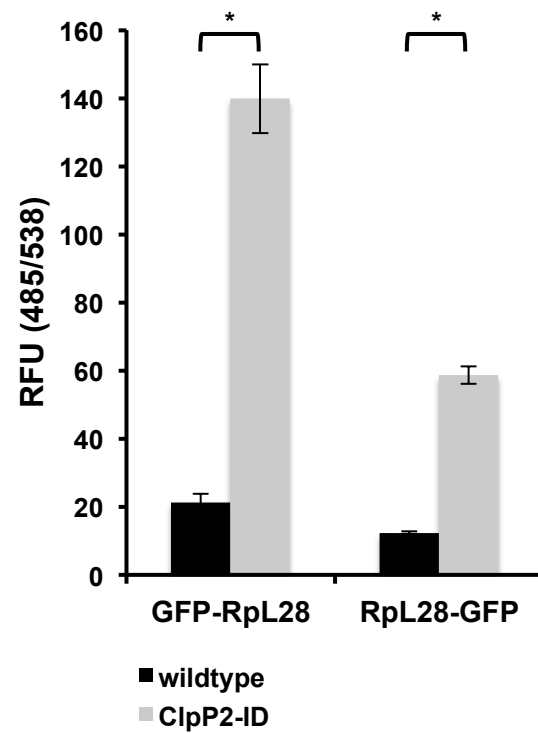**B**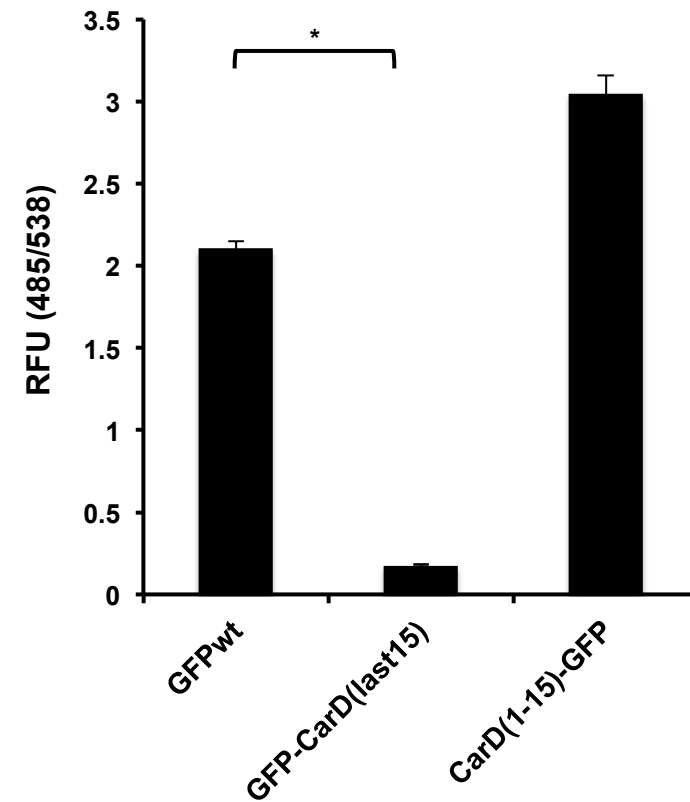

Supplement: Figure S4 — Characterization of DnaA, RpL28 as potential substrates of Clp protease, and localization of CarD degron. (A) N- and C-terminal GFP-fusions for DnaA and RpL28, identified as potential Clp substrates from proteomic profiling of clpP2-ID Msm. Fluorescence (485/538) was measured for N- and C-terminal GFP fusions constructed for DnaA (left) and RpL28 (right), and induced for 8 hours in wildtype or clpP2-ID Msm with ATc (100 ng/mL). In clpP2-ID, ATc simultaneously induced fusion protein production and degradation of ClpP2. (B) Fluorescence (485/538) was measured for wildtype GFP, and GFP fusions bearing either the N-terminal C-terminal 15 amino acids from CarD. The constructs were expressed on a constitutive, episomal plasmid in wildtype Msm. In both (A) and (B), data are represented as mean RFU +/− standard deviation of biological replicates. Asterisks denote a p-value <0.05 determined by t-test. (PDF) [file ppat.1003994.s004.pdf]
