# Supplementary material for: Post-Translational Regulation via Clp Protease Is Critical for Survival of Mycobacterium tuberculosis
Source: PLoS Pathog. 2014 Mar 6;10(3):e1003994. doi: 10.1371/journal.ppat.1003994 (PMC3946367; doi:10.1371/journal.ppat.1003994)
Supplement: Table S4 — Plasmids used in this study. All plasmids used for experiments are listed, with specific references to particular plasmids included in the Supplementary Methods S1. (DOC) [file ppat.1003994.s008.doc]

**TABLE S4. Plasmids used in this study**

| **Plasmid** | **Properties/Uses** |
| --- | --- |
| **pNIT(zeo)::RecET-SacB** | Nitrile inducible plasmid to make Mtb competent for recombineering |
| **pEXPR(kan)::RT38-p750-clpP1P2DAS** | L5 integrating plasmid used to construct clpP1P2 merodiploid Mtb |
| **pGMCtKq22(zeo)::TSC10M1-pUV15-sspB** | ATc-inducible expression of sspB for DAS inducible degradation of ClpP2-DAS |
| **pTetOR(zeo)::gfp-attR** | Gateway destination vector for N-terminal GFP tagging |
| **pTetOR(zeo)::attR-gfp** | Gateway destination vector for C-terminal GFP tagging |
| **pTetOR(zeo)::gfp-carD** | ATc-inducible expression of GFP-CarD |
| **pTetOR(zeo)::gfp-whiB1** | ATc-inducible expression of GFP-WhiB1 |
| **pTetOR(zeo)::gfp-rpL28** | ATc-inducible expression of GFP-RpL28 |
| **pTetOR(zeo)::gfp-dnaA** | ATc-inducible expression of GFP-DnaA |
| **pTetOR(zeo)::carD-gfp** | ATc-inducible expression of CarD-GFP |
| **pTetOR(zeo)::whiB1-gfp** | ATc-inducible expression of WhiB1-GFP |
| **pTetOR(zeo)::rpL28-gfp** | ATc-inducible expression of RpL28-GFP |
| **pTetOR(zeo)::dnaA-gfp** | ATc-inducible expression of DnaA-GFP |
| **pMV762(zeo)::gfp-whiB1(last15)** | Constitutive expression of GFP-WhiB1(last 15 amino acids) |
| **pMV762(zeo)::gfp-whiB1(last9)** | Constitutive expression of GFP-WhiB1(last 9 amino acids) |
| **pMV762(zeo)::gfp-whiB1(last5)** | Constitutive expression of GFP-WhiB1(last 5 amino acids) |
| **pMV762(zeo)::gfp-whiB1(last3)** | Constitutive expression of GFP-WhiB1(last 3 amino acids) |
| **pMV762(zeo)::gfp-carD(last15)** | Constitutive expression of GFP-CarD(last 15 amino acids) |
| **pGH1000A(hyg)::PwhiB1(500)-Luciferase** | Integrative luciferase reporter to monitor whiB1 promoter activity |
