# Supplementary material for: Post-Translational Regulation via Clp Protease Is Critical for Survival of Mycobacterium tuberculosis
Source: PLoS Pathog. 2014 Mar 6;10(3):e1003994. doi: 10.1371/journal.ppat.1003994 (PMC3946367; doi:10.1371/journal.ppat.1003994)
Supplement: Table S5 — Primers used in this study. All primers used for strain construction and qPCR are listed, with specific references to particular primers included in the Supplementary Methods S1. (DOC) [file ppat.1003994.s009.doc]

**TABLE S5. Primers used in this study**

| **Primer Name** | **Primer Sequence (5’ to 3’)** | **Use** |
| --- | --- | --- |
| **attB4-RT38F** | GGGGACAACTTTGTATAGAAAAGTTGCAGCTGGCTAGCGAGTCATG | Cloning of RT38 into multisite gateway destination vector (forward) |
| **attB1-RT38R** | GGGGACTGCTTTTTTGTACAAACTTGAATATTGGATCACGCCGCGA | Cloning of RT38 into multisite gateway destination vector (reverse) |
| **attB1-p750F** | GGGGACAAGTTTGTACAAAAAAGCAGGCTGCTACCAGGCCTAGATCTGG | Cloning of p750 into multiple gateway destination vector (forward) |
| **attB2-p750R** | GGGGACCACTTTGTACAAGAAAGCTGGGTGGTGGTGCATGCGGTTGTGA | Cloning of p750 into multiple gateway destination vector (reverse) |
| **attB2-clpP1P2DASF** | GGGGACAGCTTTCTTGTACAAAGTGGGAAGGAGATATACCTGTGAGCCAAGTGACTGAC | Cloning of clpP1P2DAS into multiple gateway destination vector (forward) |
| **attB3-DASR** | GGGGACAACTTGTATAATAAAGTTGTCACTAGCTGGCGTCCGCGTAGTTCTCGGAGTAGT | Cloning of clpP1P2DAS into multiple gateway destination vector (round 2, reverse) |
| **clpP1P2DASR** | GTAGTTCTCGGAGTAGTTCTCGTCGTTGGCGGCGGCGGTTTGCGCGGAGAGCTTC | Cloning of clpP1P2DAS into multiple gateway destination vector (round 1, reverse) |
| **5flankF** | GCTCGTCACGCGCGGCGTT | Cloning of upstream fragment for stitch PCR to generate recombineering product to delete clpP1P2 operon (forward) |
| **5flankR** | GGGGAGTATAACTTGGGGCACCTGCTTTCCTCGA | Cloning of upstream fragment for stitch PCR to generate recombineering product to delete clpP1P2 operon (reverse) |
| **hygkoF** | AAGCAGGTGCCCCAAGTTATACTCCCCGACGTGGCC | Cloning of hygR for stitch PCR to generate recombineering product to delete clpP1P2 operon (forward) |
| **hygkoR** | TCAGGCGGTTTGCGTCTAGACTCGAGGTACCGGCG | Cloning of hygR for stitch PCR to generate recombineering product to delete clpP1P2 operon (reverse) |
| **3flankF** | CTCGAGTCTAGACGCAAACCGCCTGAGCCATGG | Cloning of downstream fragment for stitch PCR to generate recombineering product to delete clpP1P2 operon (forward) |
| **3flankR** | ATGCTCAATCTGCAGCGGTCGC | Cloning of downstream fragment for stitch PCR to generate recombineering product to delete clpP1P2 operon (reverse) |
| **clpPkoF** | GGAAGCTCAGGTTACCGTCA | Primer to check deletion of clpP1P2 operon in Mtb (forward) |
| **hygR** | GCGTAGGAATCATCCGAATC | Primer to check deletion of clpP1P2 operon in Mtb (forward) |
| **RMR257** | AAACCCTTAATTAAGAAGGAGATATACCTATGGCTA | Cloning of gfp for stitch PCR to generate N-terminal GFP Gateway tagging insert (forward) |
| **RMR258** | ACAAACTTGTTTTGTATAGTTCATCCATGCCATG | Cloning of gfp for stitch PCR to generate N-terminal GFP Gateway tagging insert (reverse) |
| **RMR259** | AACTATACAAAACAAGTTTGTACAAAAAAGCT | Cloning of attR ccdB-CM^R^ cassette for stitch PCR to generate N-terminal GFP Gateway tagging insert (forward) |
| **RMR260** | TTTTTTGATATCACCACTTTGTACAAGAAAGCTGAAC | Cloning of attR ccdB-CM^R^ cassette for stitch PCR to generate N-terminal GFP Gateway tagging insert (reverse) |
| **RMR261** | AAACCCTTAATTAAACAAGTTTGTACAAAAAAGCT | Cloning of attR ccdB-CM^R^ cassette for stitch PCR to generate C-terminal GFP Gateway tagging insert (forward) |
| **RMR262** | CCTTTGCTAGCAACCACTTTGTACAAGAAAGCTGAAC | Cloning of attR ccdB-CM^R^ cassette for stitch PCR to generate C-terminal GFP Gateway tagging insert (reverse) |
| **RMR263** | CAAAGTGGTTGCTAGCAAAGGAGAAGAACTT | Cloning of gfp for stitch PCR to generate C-terminal GFP Gateway tagging insert (forward) |
| **RMR264** | TTTTTTGATATCTCATTTGTATAGTTCATCCATGCCAT | Cloning of gfp for stitch PCR to generate C-terminal GFP Gateway tagging insert (reverse) |
| **RMR271** | GGGGACAAGTTTGTACAAAAAAGTTGCCCATATTTTCAAGGTCGGAGACACCGT | attB Gateway carD PCR product for N-terminal GFP tagging (forward) |
| **RMR272** | GGGGACCACTTTGTACAAGAAAGCTGGGTCTCAAGACGCGGCGGCTAAAAC | attB Gateway carD PCR product for N-terminal GFP tagging (reverse) |
| **RMR273** | GGGGACAAGTTTGTACAAAAAAGTTGCCCATGAAGGAGATATACCTATGATTTTCAAGGTCGGAGACAC | attB Gateway carD PCR product for C-terminal GFP tagging (forward) |
| **RMR274** | GGGGACCACTTTGTACAAGAAAGCTGGGTCAGACGCGGCGGCTAAAACCTC | attB Gateway carD PCR product for C-terminal GFP tagging (reverse) |
| **RMR275** | GGGGACAAGTTTGTACAAAAAAGTTGCCCATGATTGGCGCCACAAGGCGGT | attB Gateway whiB1 PCR product for N-terminal GFP tagging (forward) |
| **RMR276** | GGGGACCACTTTGTACAAGAAAGCTGGGTCTCAGACCCCGGTACGGGCTTTC | attB Gateway whiB1 PCR product for N-terminal GFP tagging (reverse) |
| **RMR277** | GGGGACAAGTTTGTACAAAAAAGTTGCCCATGAAGGAGATATACCTATGGATTGGCGCCACAAGGC | attB Gateway whiB1 PCR product for C-terminal GFP tagging (forward) |
| **RMR278** | GGGGACCACTTTGTACAAGAAAGCTGGGTCGACCCCGGTACGGGCTTTCG | attB Gateway whiB1 PCR product for C-terminal GFP tagging (reverse) |
| **RMR279** | GGGGACAAGTTTGTACAAAAAAGTTGCCCATTCCGCCCACTGCCAAGTCAC | attB Gateway rpL28 PCR product for N-terminal GFP tagging (forward) |
| **RMR280** | GGGGACCACTTTGTACAAGAAAGCTGGGTCTCAGATCCGCTGCCCCTGG | attB Gateway rpL28 PCR product for N-terminal GFP tagging (reverse) |
| **RMR281** | GGGGACAAGTTTGTACAAAAAAGTTGCCCATGAAGGAGATATACCTTTGTCCGCCCACTGCCAAGT | attB Gateway rpL28 PCR product for C-terminal GFP tagging (forward) |
| **RMR282** | GGGGACCACTTTGTACAAGAAAGCTGGGTCGATCCGCTGCCCCTGGCGA | attB Gateway rpL28 PCR product for C-terminal GFP tagging (reverse) |
| **RMR283** | GGGGACAAGTTTGTACAAAAAAGTTGCCCATACCGATGACCCCGGTTCAGGC | attB Gateway dnaA PCR product for N-terminal GFP tagging (forward) |
| **RMR284** | GGGGACCACTTTGTACAAGAAAGCTGGGTCCTAGCGCTTGGAGCGCTGAC | attB Gateway dnaA PCR product for N-terminal GFP tagging (reverse) |
| **RMR285** | GGGGACAAGTTTGTACAAAAAAGTTGCCCATGAAGGAGATATACCTTTGACCGATGACCCCGGTTCA | attB Gateway dnaA PCR product for C-terminal GFP tagging (forward) |
| **RMR286** | GGGGACCACTTTGTACAAGAAAGCTGGGTCGCGCTTGGAGCGCTGACGG | attB Gateway dnaA PCR product for C-terminal GFP tagging (reverse) |
| **RMR248** | AAAAAAATTAATTAAGAAGGAGATATACCTATGGCTA | Cloning of gfp truncations with variable number of 3’ base pairs from whiB1 and carD (forward) |
| **RMR249** | AAAAAAAATCGATTCAGACCCCGGTACGGGCTTTCGTGCGGGCGTTGCGACGCTTCAGTTTGTATAGTTCATCCATGCCA | Cloning of gfp-whiB1[last 15 amino acids] (reverse) |
| **RMR294** | AAAAAAAATCGATTCAGACCCCGGTACGGGCTTTCGTGCGGGCTTTGTATAGTTCATCCATGCCA | Cloning of gfp-whiB1[last 9 amino acids] (reverse) |
| **RMR295** | AAAAAAAATCGATTCAGACCCCGGTACGGGCTTTGTATAGTTCATCCATGCCA | Cloning of gfp-whiB1[last 5 amino acids] (reverse) |
| **RMR296** | AAAAAAAATCGATTCAGACCCCGGTTTTGTATAGTTCATCCATGCCA | Cloning of gfp-whiB1[last 3 amino acids] (reverse) |
| **RMR298** | AAAAAAAATCGATTCAAGACGCGGCGGCTAAAACCTCGTCAAGGATGGTCTCGGCTTTGGCTTTGTATAGTTCATCCATGCCA | Cloning of gfp-carD[last 15 amino acids] (reverse) |
| **RMR313** | AAAAAAATCTAGAGGGGTGTTTGCGACGACCAG | Cloning of PwhiB1(500 bp) for stitch PCR to create luciferase promoter reporter (forward) |
| **RMR310** | GGCGTCTTCCATGTGATCTAACTCCTAATCGGGCGC | Cloning of PwhiB1(500 bp) for stitch PCR to create luciferase promoter reporter (reverse) |
| **RMR311** | AGGAGTTAGATCACATGGAAGACGCCAAAAACATAAAGAAAGG | Cloning of luciferase for stitch PCR to create luciferase promoter reporter (reverse) |
| **RMR312** | AAAAAAAAAGCTTCTATTTCACGGCGATCTTTCCGC | Cloning of luciferase for stitch PCR to create luciferase promoter reporter (reverse) |
