# Supplementary material for: Post-Translational Regulation via Clp Protease Is Critical for Survival of Mycobacterium tuberculosis
Source: PLoS Pathog. 2014 Mar 6;10(3):e1003994. doi: 10.1371/journal.ppat.1003994 (PMC3946367; doi:10.1371/journal.ppat.1003994)
Supplement: Methods S1 — Supplementary Methods S1 includes further information on (1) Bacterial strains and plasmids, (2) Sample fractionation by high pH reverse phase and strong cation exchange chromatography, (3) Liquid chromatography electrospray ionization tandem mass spectrometry, and (4) Data processing: MS2 spectra assignment, data filtering and quantitative data analysis. (DOC) [file ppat.1003994.s010.doc]

**SUPPLEMENTAL MATERIALS AND METHODS S1.**

**Bacterial strains and plasmids**

The construction of strain clpP2_ID Msm has been described in depth elsewhere. The first step in the construction of P750-clpP1P2DAS was transformation of the attB L5 site integrating plasmid, pEXPR(kan)::RT38-p750-P1P2DAS into H37Rv/pNIT(zeo)::RecET-SacB to create a clpP1P2 merodiploid. The pEXPR(kan) plasmid was generated using multi-site gateway to insert the tetracycline-off repressor (RT38, PCR amplified using primers: attB4-RT38F, attB1-RT38R), the tetracycline-off promoter (p750, PCR amplified using primers: attB1-p750F, attB2-p750R), and the DAS-modified clpP1P2 operon (P1P2DAS, 2 rounds of PCR used to append DAS tag using primers: attB2-clpP1P2DASF, clpP1P2DASR, attB3-DASR) into the multisite gateway entry vector, pEN23A(kan). The DAS modification, which appends the DAS tag to the 3’-end of clpP2, was inserted to facilitate inducible degradation of ClpP2 upon expression of SspB. The plasmidpNIT(zeo)::RecET-SacB, which contains the machinery for mycobacterial recombineering, was a gift from the laboratory of Chris Sassetti. Next, hygromycin-containing knockout-out fragments were generated using stitch PCR to ligate 500 bp upstream (primers: 5flankF, 5flankR) and downstream (primers: 3flankF, 3flankR)of the clpP1P2 operonto a hygromycin resistance marker (primers: hygkoF, hygkoR). This linear PCR fragment was transformed into the above Mtb strain that was made competent for recombineering. This was done by addition of 1 mMisovaleronitrile(IVN, Sigma Aldrich) to a culture at OD_600_ 0.8. IVN addition induced expression of the recombineering machinery on pNit(zeo)::RecET-SacB. After 8h, 10 mL of 2M glycine was added, and the culture was grown overnight to yield recombineering-competent Mtb. Electroporation was performed as previously described[1]. Positive clones were plated on 7H10 agar containing 10% sucrose to counterselect against the recombinase plasmid, and scored for growth on zeocin-containing agar to confirm the loss of pNit::RecET. Deletion of the endogenous clpP1P2 operon was confirmed by PCR screening using primers that annealed outside of the region deleted and to the hygromycin resistance marker (primers:clpPkoF, hygR). In a final step, the tweety site integrating plasmid, pGMCtKq22(zeo)::TSC10M1-pUV15-sspB, was transformed into the strain. This plasmid, a gift from Dirk Schnappinger, inducibly expressed SspB and enabled degradation of the DAS-modified ClpP2 protein,

To construct GFP whole protein fusions, tetracycline-inducible, Gateway destination vectors were generated that enabled either N- or C-terminal fusion of an Mtb open reading frame to GFP. For N-terminal tagging, stitch PCR was used to append GFP, with a ribosome binding site upstream of the translational start site, (primers: RMR257, RMR258) upstream of an attRccdB-Cm^R^cassette (primers: RMR259, RMR260). For C-terminal tagging, stich PCR was used to append GFP (primers: RMR263, RMR264) downstream of an attRccdB-CM^R^ cassette (primers: RMR261, RMR262). Both products were digested with PacI and EcoRV and then ligated into the tetracycline-inducible episomal pTetOR(zeo) plasmid, a gift from Mike Chao, to yield the final destination vectors. For N-terminal GFP tagging to carD, whiB1, rpL28, and dnaA, genes were amplified using primers containing attB sites (primers: RMR271,RMR272 [carD]; RMR275, RMR276 [whiB1]; RMR279, RMR280 [rpL28]; RMR283, RMR284 [dnaA]). The only difference for C-terminal tagging of Mtb genes was that the 5’ end primer contained a ribosome-binding site between the attB1 sequence and ATG start of the gene (primers: RMR273, RMR274 [carD], RMR277, RMR278 [whiB1], RMR281, RMR282 [rpL28], RMR285, RMR286 [dnaA]).

The constitutively expressing GFP fusions, bearing the truncated C-termini of WhiB1 or CarD were generated using primers to amplify GFP, in which the reverse complement primer contained 3’ end bases of carD or whiB1 (primers: RMR248, RMR249, RMR294, RMR295, RMR296, RMR298). These modified gfp PCR products were then digested with PacI and ClaI and ligated into the episomal plasmid pMV762(zeo), a gift from Meera Unnikrishnan, in which inserted products were regulated by the constitutive groEL promoter.

The luciferase reporter to monitor whiB1 promoter activity was generated using stich PCR to ligate the 500 bp upstream of whiB1 (primers: RMR313, RMR310) to the firefly luciferase gene (primers: RMR311, RMR312). This fused construct was then digested with XbaI and HindII and ligated into pGH100A(hyg), a promoter-less plasmid that integrates into the chromosome at the Giles integration site.

**Sample fractionation by high pH reverse phase and strong cation exchange chromatography**

Fractionation of Mtb TMT-labeled peptides was performed by high pH reverse phase liquid chromatography. Briefly, the sample was resuspended in high pH buffer A (10 mM ammonium formate pH 8.5, 5% acetonitrile), and separated over a 4.6 X 250 mm 300 Extend C18 analytical HPLC column (5 μm, 300 Å, Agilent). Separation involved a two-buffer (high pH buffers A and B) gradient from 0% to 20% high pH buffer B (10 mM ammonium formate pH 8.5, 96% acetonitrile) in 5 min at a flow rate of 0.8 ml min-1, followed by 20% to 45% high pH buffer B in 50 min at a flow rate of 0.8 ml min-1, followed by 45 to 100% high pH buffer B in 4 min at a flow rate of 0.8 ml min-1 using an Agilent 1100 quaternary pump outfitted with a degasser and a photodiode array detector (PDA) (Thermo Scientific). Samples were collected in 30-s increments into a 96-well plate and dried under vacuum. For even columns in the 96 well plate, wells A,C,E,G were combined into one fraction, while for odd columns, wells B,D,F,H were combined, resulting in 12 total fractions, which were dried under vacuum. Fractions were then redissolved with 1% formic acid, desalted by StageTips and re-dried under vacuum.

Fractionation of Msm TMT-labeled peptides was performed by SCX chromatography. Briefly, the sample was resuspended in SCX chromatography buffer A (7 mM KH2PO4 (pH 2.6) and 30% acetonitrile) and separated over a 4.6 mm × 200 mm polysulfoethyl A HPLC column (5 μm, 200 Å, PolyLC). Separation involved a two-buffer (SCX chromatography buffers A and B) gradient from 0 to 50% SCX chromatography buffer B (7 mM KH2PO4, 350 mMKCl (pH 2.6) and 30% acetonitrile) in 47 min at a flow rate of 0.9 ml min−1, followed by 50 to 100% SCX chromatography buffer A to buffer B in 4.5 min using an Agilent 1100 quaternary pump outfitted with a degasser and a photodiode array detector (PDA) (Thermo Scientific). Samples were collected in 30-s increments into a 96-well plate and dried under vacuum. Fractions were then redissolved with 1% formic acid and, based on the intensity from the SCX chromatographic UV-light trace, combined into a total of 20 samples of similar peptide amount, which were desalted by Stagetips and dried under vacuum.

**Liquid chromatography electrospray ionization tandem mass spectrometry**

All proteomic methodology was adapted from a methods paper published on comparing MS2 to MS3 based quantitative proteomics using TMT isobaric labeling[2]. Briefly, all LC-MS2(MS3) experiments were performed on an LTQ OrbitrapVelos (Thermo Fischer Scientific) equipped with a Famos autosampler (LC Packings) and an Agilent 1100 binary high-pressure liquid chromatography (HPLC) pump (Agilent Technologies). Peptides were separated on a 100 μm inner diameter microcapillary column packed first with approximately 0.5 cm of Magic C4 resin (5 μm, 100 Å, Michrom Bioresources) followed by 20 cm of Maccel C18AQ resin (3 μm, 200 Å, Nest Group). Separation was achieved by applying a 9–32% acetonitrile gradient in 0.125% formic acid over 150 min at ~300 nl min−1. Electrospray ionization was enabled through applying a voltage of 1.8 kV through a polyetheretherketone (PEEK) junction at the inlet of the microcapillary column.

The LTQ Orbitrap Velos was operated in data-dependent mode for both MS2 and MS3 methods. For the MS2 method, the survey scan was performed in the Orbitrap in the range of 300–1,500 *m/z* at a resolution of 3 × 10^4^, followed by the selection of the ten most intense ions (top 10) for HCD-MS2 fragmentation using a precursor isolation width window of 2 *m/z*. The automatic gain control (AGC) settings were 3 × 10^6^ ions and 2.5 × 10^5^ ions for survey and MS2 scans, respectively. Ions were selected for MS2 when their intensity reached a threshold of 500 counts and an isotopic envelope was assigned. Maximum ion accumulation times were set to 1,000 ms for survey MS scans and to 250 ms for MS2 scans. The normalized collision energy for HCD-MS2 experiments was set to 45% at a 30-ms activation time. Singly charged ion species and ions for which a charge state could not be determined were not subjected to MS2. Ions within a 10 p.p.m. *m/z* window around ions selected for MS2 were excluded from further selection for fragmentation for 120 s.

The survey MS scan settings were identical for the MS3 method, where the ten most intense ions were first isolated for ion trap CID-MS2 at a precursor ion isolation width of 2 *m/z*, using an AGC setting of 2 × 10^3^, a maximum ion accumulation time of 150 ms and wide band activation. Directly after each MS2 experiment, the most intense fragment ion in an *m/z* range between 110–160% of the precursor *m/z* was selected for HCD-MS3. The fragment-ion isolation width was set to 4 *m/z*, the MS3 AGC was 20 × 10^4^ and the maximum ion time 250 ms. We chose an isolation width of 4 *m/z* to avoid space charging in preliminary experiments with very high AGC settings for the MS3 scan; but with the AGC settings used in the experiments described here, setting 2 or 4 *m/z* as the MS3 isolation width had a negligible effect on the results (data not shown). Normalized collision energy was set to 35% and 60% at an activation time of 20 ms and 50 ms for MS2 and MS3 scans, respectively. It is important to note that charge state screening has to be disabled to allow fragment ions to be selected for MS3; this setting nevertheless allows for charge state-based exclusion of singly charged ions and ions for which no charge state was determined from MS2.

**Data processing: MS2 spectra assignment, data filtering and quantitative data analysis**

A suite of in-house–developed software tools was used to convert mass spectrometric data from the RAW file to the mzXML format, as well as to correct erroneous assignments of peptide ion charge state and monoisotopic *m/z*. We modified the ReAdW.exe to include ion accumulation time in the output during conversion to the mzXML file format (http://sashimi.svn.sourceforge. net/viewvc/sashimi/). Assignment of MS2 spectra was performed using the Sequest algorithm by searching the data against a protein sequence database containing all known translated proteins from either Mtb or Msm, and known contaminants such as porcine trypsin, and human keratin[3]. This forward (target) database component was followed by a decoy component including all listed protein sequences in reversed order. Searches were performed using a 50 p.p.m. precursor ion tolerance, where both peptide termini were required to be consistent with Lys-C specificity, while allowing up to two missed cleavages. Sixplex TMT tags on lysine residues and peptide N termini (+ 229.162932 Da) and carbamido- methylation of cysteine residues (+57.02146 Da) were set as static modifications, oxidation of methionine residues (+ 15.99492 Da) as a variable modification. An MS2 spectral assignment false discovery rate of less than 1% was achieved by applying the target- decoy database search strategy[4]. Filtering was performed using a linear discrimination analysis method to create one combined filter parameter from the following peptide ion and MS2 spectra properties: Sequest parameters XCorr and ∆Cn, peptide ion mass accuracy and charge state, predicted low pH (2.7) in-solution charge of peptide and peptide length. Linear discrimination scores were used to assign probabilities to each MS2 spectrum for being assigned correctly and these probabilities were used to filter the dataset with an MS2 spectra assignment false discovery rate of less than 1% to obtain a protein identification false discovery rate of less than 1.5%[5].

For quantification, a 0.06 *m/z* window centered on the theoretical *m/z* value of each reporter ion was monitored for ions, and the intensity of the signal closest to the theoretical *m/z* value was recorded. Reporter ion intensities were denormalized by multiplication with the ion accumulation time for each MS2 or MS3 spectrum and adjusted based on the overlap of isotopic envelopes of all reporter ions. Intensity distributions of isotopic envelopes were as provided by the manufacturer. The total signal intensity across all peptides quantified were summed for each TMT channel, and all intensity values were normalized to account for potentially uneven TMT labeling. For MS3-based Mtb proteomics, the intensities for all peptides of a given protein were summed to derive an overall protein abundance value for each TMT signal. For MS2-based Msm proteomics, the overall protein abundance was calculated by taking the median intensity value of all peptides of that protein within each TMT channel. Hierarchical clustering using Pearsons correlational analysis was conducting using MultiExperiment Viewer (TM4 Microarray Software Suite). Statistical significance between replicates was determined using an unpaired t-test grouping biological replicates.

1. van Kessel JC, Hatfull GF (2006) Recombineering in Mycobacterium tuberculosis. Nat Methods 4: 147–152. doi:10.1038/nmeth996.

2. Ting L, Rad R, Gygi SP, Haas W (2011) MS3 eliminates ratio distortion in isobaric multiplexed quantitative proteomics. Nat Methods 8: 937–940. doi:10.1038/nmeth.1714.

3. Eng J, McCormack A, Yates J III (1994) An approach to correlate tandem mass spectral data of peptides with amino acid sequences in a protein database. Journal of the American Society for Mass Spectrometry 5: 976–989.

4. Elias J, Gygi S (2007) Target-decoy search strategy for increased confidence in large-scale protein identifications by mass spectrometry. Nat Methods 4: 207–214.

5. Huttlin EL, Jedrychowski MP, Elias JE, Goswami T, Rad R, et al. (2010) A Tissue-Specific Atlas of Mouse Protein Phosphorylation and Expression. Cell 143: 1174–1189. doi:10.1016/j.cell.2010.12.001.
